# Fatal Dengue Cases Reveal Brain Injury and Viral Replication in Brain-Resident Cells Associated with the Local Production of Pro-Inflammatory Mediators

**DOI:** 10.3390/v12060603

**Published:** 2020-05-31

**Authors:** Natália Salomão, Kíssila Rabelo, Carlos Basílio-de-Oliveira, Rodrigo Basílio-de-Oliveira, Luiz Geraldo, Flávia Lima, Flávia dos Santos, Gerard Nuovo, Edson R. A. Oliveira, Marciano Paes

**Affiliations:** 1 Interdisciplinary Medical Research Laboratory Rio de Janeiro, Oswaldo Cruz Foundation, 21040-900 Rio de Janeiro, Brazil; natgsalomao@gmail.com; 2 Ultrastructure and Tissue Biology Laboratory Rio de Janeiro, Rio de Janeiro State University, 20551-030 Rio de Janeiro, Brazil; kissilarabelo91@gmail.com; 3 Pathological Anatomy, Gaffrée Guinle University Hospital Rio de Janeiro, Federal University of the State of Rio de Janeiro, 20270-004 Rio de Janeiro, Brazil; basiliopatologia@br.inter.net (C.B.-d.-O.); rodrigopboliveira@gmail.com (R.B.-d.-O.); 4 Glial Cell Biology Laboratory, Institute of Biomedical Sciences Rio de Janeiro, Federal University of Rio de Janeiro, 21941-590 Rio de Janeiro, Brazil; lh_geraldo@hotmail.com (L.G.); flima@icb.ufrj.br (F.L.); 5 Viral Immunology Laboratory, Oswaldo Cruz Institute Rio de Janeiro, Oswaldo Cruz Foundation, 21040-900 Rio de Janeiro, Brazil; flaviabarretod@gmail.com; 6 Ohio State University Comprehensive Cancer Center, Ohio State University Foundation, Columbus, OH 43210, USA; jerrynuovo@yahoo.com; 7 Phylogeny Medical Laboratory Columbus, Ohio State University Foundation, Columbus, OH 43214, USA; 8 Department of Microbiology and Immunology Chicago, University of Illinois at Chicago, Chicago, IL 60612, USA; edsonrao@gmail.com

**Keywords:** dengue, human fatal cases, neuropathogenesis, inflammation, central nervous system

## Abstract

Dengue is an arboviral disease caused by dengue virus (DENV), which is transmitted to humans by *Aedes aegypti* mosquitoes. Infection by DENV most commonly results in a mild flu-like illness; however, the disease has been increasingly associated with neurological symptomatology. This association draws attention to further investigations on the impact of DENV infection in the host’s central nervous system. Here, we analyzed brain samples of three fatal dengue cases that occurred in 2002 during an outbreak in Rio de Janeiro, Brazil. Brain tissues of these cases were marked by histopathological alterations, such as degenerated neurons, demyelination, hemorrhage, edema, and increased numbers of astrocytes and microglial cells. Samples were also characterized by lymphocytic infiltrates mainly composed of CD8 T cells. DENV replication was evidenced in neurons, microglia and endothelial cells through immunohistochemistry and in situ hybridization techniques. Pro-inflammatory cytokines, such as TNF-α and IFN-γ were detected in microglia, while endothelial cells were marked by the expression of RANTES/CCL5. Cytoplasmic HMGB1 and the production of nitric oxide were also found in neurons and microglial cells. This work highlights the possible participation of several local pro-inflammatory mediators in the establishment of dengue neuropathogenesis.

## 1. Introduction

Dengue is a mosquito-borne viral disease that mainly occurs in tropical and sub-tropical regions of the world. Dengue is a mild flu-like illness caused by the infection with one of the four viral serotypes, DENV1 to DENV4, which belong to the *Flaviviridae* family. It is estimated that nearly 390 million people are infected with DENV every year, of which 96 million are symptomatic [1]. A small percentage of infected patients (1–5%) evolve to severe dengue, which is a life-threatening complication characterized by plasma leakage, fluid accumulation, respiratory distress, severe bleeding, and organ impairments [2]. The circumstances that favor the progression from a mild to a severe case are not fully understood. However, it is known that biological factors, such as virus strain and the status of host’s immunity can contribute to detrimental progression [3,4], mostly in secondary infections [5,6,7,8]. The majority of severe dengue cases are developed after virus clearance, which suggests that the severe disease occurs under an immunopathological process.

The involvement between the infection and the host’s central nervous system (CNS) has drawn attention in dengue. Classically, CNS symptoms in a dengue context are seen as uncommon in humans [9,10,11]. An increasing number of studies showing the presence of DENV in the host’s CNS [12,13,14,15] have suggested the contribution of the virus in generating CNS-related manifestations. In the last decades, several reports have shown that these manifestations can be of encephalopathic, neuromuscular or neuro-ophthalmic nature [7,10,11,16,17,18,19,20,21,22]. Neurological manifestations in dengue can also be highly subjective and involve symptoms of restlessness, irritability, dizziness, and drowsiness [4]. Given this scenario, neurological manifestations are now officially recognized in severe dengue by the World Health Organization (WHO) [23]. However, the precise mechanisms of how dengue neuropathogenesis takes place are still unknown.

In recent years, our group has been investigating fatal dengue cases as a strategy to gain knowledge on the pathogenesis of the disease. In this context, we reported histopathological and ultrastructural alterations caused by DENV, as well as systemic viral spread [24]. Moreover, we observed that fatal dengue cases presented relevant local pro-inflammatory responses in their peripheral organs, with the participation of IFN-γ, TNF-α and RANTES/CCL5-producing cells [25,26]. Aiming to better understand the impact of DENV infection in the host’s CNS, here, we extended our analysis towards the brain environment of these fatal cases. In this regard, we found that brain samples were marked by histological alterations, such as circulatory dysfunction and degenerated neurons. DENV antigen was detected within different cell types in the brain, indicating that the virus holds neurotrophic properties. The viral presence in the brain was associated with the altered morphology of glial cells, such as microglia and astrocytes. Local host response was marked by the production of an array of pro-inflammatory markers, such as TNF-α, IFN-γ, RANTES/CCL5, and nitric oxide (NO). DENV-specific high mobility group box 1 (HMGB1) response was also characterized within the CNS environment of fatal dengue cases. This work highlights a possible participation of several pro-inflammatory mediators in the development of CNS-related symptoms upon DENV infection.

## 2. Materials and Methods 

### 2.1. Ethical Procedures

All procedures performed during this work with fatal dengue cases and controls were approved by the Ethics Committee of the Oswaldo Cruz Foundation/FIOCRUZ (CAEE: 47525115.3.0000.5248). All the experimental protocols used herein were also approved by the same institutional committee mentioned above. Informed consent was obtained from all subjects.

### 2.2. Human Fatal Cases

Brain samples used in this study were extracted from fatal DENV cases described above, which occurred in Rio de Janeiro, Brazil in 2002. The Secretary of Health Surveillance sent the samples to Flavivirus Laboratory, Oswaldo Cruz Institute to perform confirmatory dengue tests. The non-dengue cases used in this work were also tested in Flavivirus Laboratory, with IgM results negative for dengue.

Case 1: Female, 21 years old, presented fever, myalgia and headache for 8 days. Characterized also with metrorrhagia, nausea, vomiting and diarrhea. The patient presented severe leukopenia and thrombocytopenia with platelet counts of 10,000/mm^3^. The patient was admitted in the Intensive care unit (ICU) of Clementino Fraga Filho University Hospital presenting respiratory failure, followed by multiple organ failure and refractory shock. There were no fresh samples or serum sample, only formaldehyde samples. Immunohistochemistry (*in house*) was positive for dengue in paraffin liver sample.

Case 2: Female, 41 years old, admitted to Miguel Couto Hospital showing weakness, fainting, sweating, epigastric pain, fever, abdominal pain, hematocrit of 48% and fluid in the abdominal cavity. The patient also presented encephalitis of probable viral etiology. After being diagnosed with dengue hemorrhagic fever the patient died from an acute pulmonary edema. IgM ^*1^ was positive for dengue; NS1 antigen detection ^*2^ and RT-PCR DENV-3 ^*3^ positive in fresh samples in the brain, lung, liver and kidney.

Case 3: Female, 61 years old, hospitalized in Miguel Couto ICU, with dengue symptoms such as fever, myalgia, vomiting and diarrhea. The patient also presented mild cerebral edema, and died from acute pulmonary edema with sudden cardiac arrest. RT-PCR DENV-3 ^*2^ was positive in fresh samples of spleen, brain, lung, liver and kidney.

*1 Kuno et al. protocol [27]
*2 PlateliaTM Dengue NS1 Ag. Kit (Bio-Rad)
*3 Lanciotti et al. protocol [28]

### 2.3. Histopathological Analysis

The histological analyses were carried out according to Paes and coworkers [6]. Briefly, fragments of brain from fatal dengue cases were fixed in 10% buffered formalin, dehydrated in ethanol, clarified in xylene and blocked in paraffin resin. In sequence, samples were sectioned in 5-mm-thick units, deparaffinized in xylene and rehydrated with alcohol. Samples were stained with hematoxylin and eosin (HE) and visualized under a light microscopy (Olympus BX 53F, Shinjuku, Japan). Digital images were rendered using Image Pro Plus software version 4.5.

### 2.4. Immunohistochemistry

For immunohistochemical analysis, the paraffin-embedded tissues (5 µm thick) were incubated for 1 h at 60 °C, deparaffinized in xylene and rehydrated with alcohol. Antigen retrieval was performed by heating the tissue in the presence of citrate buffer. Next, tissues sections were incubated with 3% hydrogen peroxidase in methanol for 10 min to block endogenous peroxidase and then, rinsed in Tris-HCl (pH 7.4). To reduce non-specific binding, sections were incubated in Protein Blocker solution (Spring Bioscience, Fremont, CA, USA) for 10 min. Samples (cuts of brain and cerebellum) were incubated with primary antibodies (anti-IBA-1: Wako, 1:200; anti-GFAP: Sigma, 1:300; anti-DENV: Produced in Swiss mice inoculated with DENV-3, 1:300; anti-NS3: Expressed in *Escherichia coli* and purified and inoculated in Balb/c mice, 1:100; anti-CD8: Dako, 1:300; anti- TNF-α: Abcam, 1:200; anti-IFN-γ: BD Bioscences, 1:100; anti-RANTES: Abcam, 1:300; anti-NO: Sigma, 1:300 and anti-HMGB-1: Abcam, 1:500) at 4 °C overnight. The specificity of anti-NS3 antibody was confirmed by Costa and collaborators with studies with DNA vaccines based on the NS3 protein and tested in mice [29]. Next, sections were incubated for 10 min with secondary complement (REVEAL complement—Spring Bioscience, Fremont, CA, USA) and with a rabbit anti-mouse IgG-HRP conjugate (REVEAL polyvalent HRP— Spring Bioscience, Fremont CA, USA) at room temperature for 15 min. Reactions were revealed with diaminobenzidine (Spring Biosciense, Fremont, CA, USA), and the tissue sections were counterstained with Meyer’s hematoxylin (Dako, Santa Barbara, CA, USA). Sections were analyzed under an Olympus BX 53 microscope and frames were acquired using a coupled Olympus DP72 camera.

### 2.5. In-Situ Hybridization

In-situ hybridization was performed to assess the viral replication. For this, a probe (5′-TGACCATCATGGACCTCCA-3′) which anneals in a conserved region within the NS3 gene in the negative strand of viral RNA was used. This probe was tested before in a mouse model infected with DENV, in which a positive reaction was only observed in tissues from virus-infected animals [6].

Paraffin-embedded sections of dengue cases and controls (5 µm) were treated before the In-situ hybridization technique [30]. Briefly, deparaffinized sections of brain and cerebellum were digested with pepsin at 1.3 mg/mL for 30 min, incubated with the probe cocktail at 60 °C for 5 min for denaturation, followed by hybridization at 37 °C overnight. Next, samples were washed with 0.2× SSC and 2% bovine serum albumin at 55 °C for 5 min. The probe-target complexes were revealed by the activity of alkaline phosphatase conjugated to anti-digoxigenin. 

### 2.6. Detection of DENV RNA and HMGB1

Detection of dengue virus and HMGB1 was performed, as previously described [30], using in-situ hybridization and immunoperoxidase staining. First, the DENV probe was tagged with 5′ digoxigenin and locked nucleic acid (LNA) modified (Exiqon, Vedbaek, Denmark). Resulting complexes were visualized using an antidigoxigenin-alkaline phosphates conjugate and nitro-blue tetrazolium and 5-bromo-4-chloro-39-indolyphosphate as the chromogen. In sequence, detection of HMGB1 was performed by immunoperoxidase (anti-HMGB1, ABCAM ab79823) using Leica Bond Max automated platform (Leica Biosystems, Wetzlar, Germany) and DAB as the chromogen. No counterstain was done. Data were analyzed by the computer based Nuance system (Caliper Life Sciences, Hopkinton, MA, USA) which separates the different chromogenic signals, converts them to fluorescent-based signals and combine them to determine co-staining.

### 2.7. Data Availability

All data generated or analyzed during this study are included in this published article.

## 3. Results

### 3.1. Fatal Dengue Cases Present Injury in the Cerebral Cortex and White Matter

**Figure 1:**
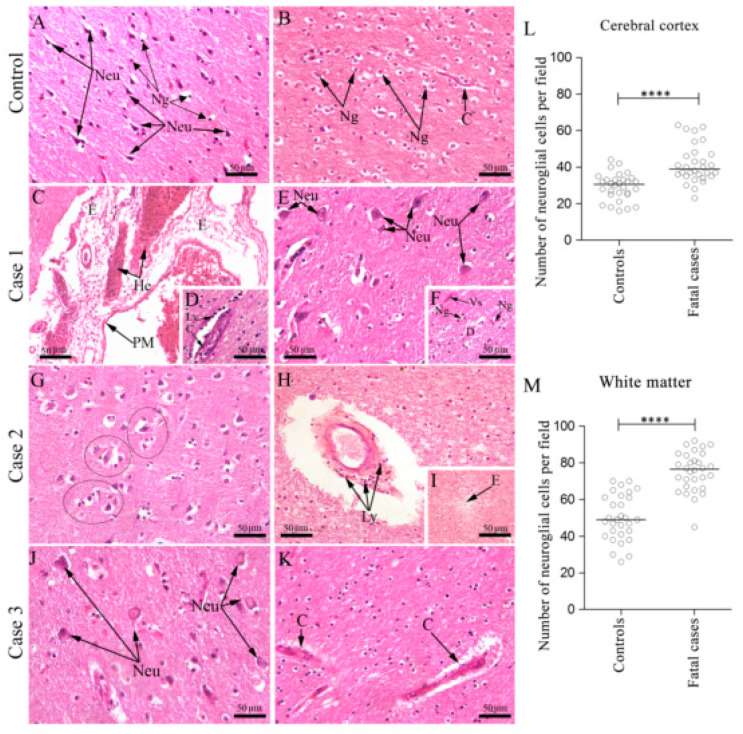
Histopathological analysis of the brain. (**A**) Cortical area of a non-dengue case with regular neurons and neuroglial cells; (**B**) White matter of a non-dengue fatal case with regular neuroglial cells and capillary; (**C**) Brain section of case 1 exhibited edema and hemorrhage areas in the pia-matter, (**D**) numerous mononuclear cells in the capillaries; (**E**) perineuronal vacuolation, (**F**) demyelination areas with presence of neuroglia cells; (**G**) Brain section of case 2 showing many “cuffs” of degenerated neurons and neuroglia cells, (**H**) lymphocytic cells infiltrate around of capillaries, (**I**) and edema; (**J**) Brain section of case 3 exhibiting intense neuronal degeneration (**K**) and thickening of the basement membrane of capillaries. (**L**,**M**) Neuroglial cells quantification assessed by Mann–Whitney U test, in the cortex and the white matter, respectively. Asterisks indicate statistically significant differences between samples: *** *p* < 0.0001. Cortical area (**A**,**E**,**G**,**J**) and white matter area (**B**–**D**,**F**,**H**,**I**,**K**). WM—White matter, Cc—Cerebral cortex, C—Capillary, PyN—Pyramidal neurons, Neu—Neuron, E—Edema, He—Hemorrhage, Ly—Lymphocytes, D—Demyelination, Vs—Vessel, Ng—Neuroglia, Black circles—“cuffs” of neurons and microglia.

In a first step, we investigated the histological aspects of brain tissues obtained from three fatal dengue cases applying hematoxylin and eosin (H.E.) staining. In this analysis we took into account two major areas of the brain: (i) the cortical region, which is the outer layer of neural tissue composed of neuronal cell bodies and neuroglial cells, that plays a key role in memory [31], attention [32], perception [33] and consciousness [34]; (ii) the white matter, which is a more internal region of the brain important in the learning process [35,36], composed of myelinated axons responsible for the transit of signals along different brain regions. Control cases obtained from individuals without commitment of the CNS had these areas with preserved structures and no relevant alterations (Figure 1A,B).

#### 3.1.1. Morphological Aspects

Case 1: This case showed commitment of structures present in the meningeal region near the pia mater, which is a more external layer to the cerebral cortex. In particular, this layer showed hemorrhagic areas associated with edema (Figure 1C). In the white matter, capillaries were marked by thickening of the basement membrane with several lymphocytes cells surrounding the vessel vein (Figure 1D). The cortical region presented perineuronal vacuolation (Figure 1E). Additionally, an increased number of neuroglial cells (neurogliosis) were found in the white matter (Figure 1F).

Case 2: Analyses of the brain cortical region in Case 2 revealed degenerated neurons forming “cuffs” with microglial cells (Figure 1G). In the white matter, blood vessel were thick, and with the presence of several surrounding lymphocytes (Figure 1H). In this case, the white matter showed several areas of edema (Figure 1I).

Case 3: Brain cuts from Case 3 were characterized by intense neuronal degeneration, with some nuclei displaced to the periphery of the neuron, in the cortical area with no relevant alterations on the neuropil (Figure 1J). In the white matter, blood vessel walls were marked by thickening and degeneration with accumulation of perivascular cells (Figure 1K).

#### 3.1.2. Quantitative Aspects

The cortex and the white matter areas were considered for quantitative analysis of glial cell numbers. Data from three non-dengue controls and the three fatal dengue cases were arranged into two distinct groups prior to comparison. The cortex region of dengue cases showed a marginal increase in the number of neuroglial cells, as compared to controls. In this case, the median of the number of neuroglial cells per field were 30 and 39, respectively, comprising an increment of nearly 30% (Figure 1L). In the white matter region, neuroglial cell numbers were more drastically increased with counts per field equal to 49 in the controls and 77 in the dengue samples, representing an increase of nearly 60% (Figure 1M). The observed differences in the neuroglial cell counts between controls and dengue samples were statistically significant. 

### 3.2. Fatal Dengue Cases Exhibit Altered Morphology of Microglial Cells and Astrocytes

**Figure 2:**
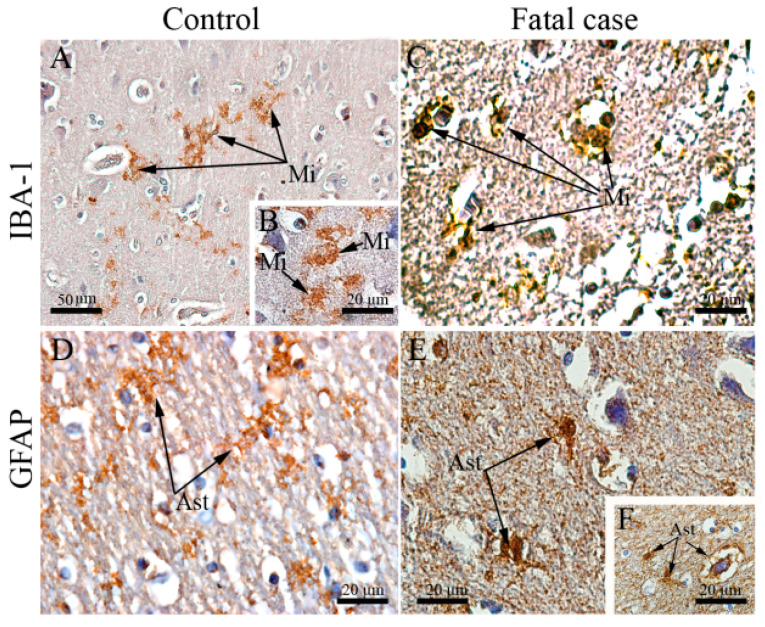
Aspects of microglia and astrocytes in brain tissue of fatal dengue cases. (**A**,**B**) Brain of a non-dengue case exhibiting microglia with normal aspect; (**C**) Brain section of DENV fatal case noting altered microglia with extensions retraction; (**D**) Brain of a non-dengue case exhibiting astrocytes with normal aspect; (**E**,**F**) Brain section of DENV fatal case showing altered astrocytes with thicker extensions. Mi—Microglia, Ast—Astrocytes.

Since we observed commitment of regular structures of the brain in the studied cases, in our next step we investigated the status of local homeostatic elements. Given the well-known role of microglial cells and astrocytes in protecting neurons from damage [37,38,39,40], we conducted a morphological analysis using specific cell markers: (i) ionized calcium-binding adaptor protein-1 (IBA-1), which is specifically and constitutively expressed in all microglial cells; (ii) glial fibrillary acidic protein (GFAP), that is found in the mature and developing astrocytes in the CNS. Microglial cells (IBA-1+) were found with activated morphology in the cortical region of the brain collected from fatal dengue cases. These cells were hyperplasic and presented with round shapes and retracted extensions reassembling an amoeboid morphology (Figure 2C). This morphological status contrasts with their typical shape that is a ramified cell with scarce cytoplasm and plasma membrane arranged with long and thin protrusions (Figure 2A,B). The astrocyte population (GFAP+ cells) also presented with altered morphology in brain cuts from fatal dengue cases. More specifically, GFAP+ cells showed thicker and less numerous extensions either in the cortical area (Figure 2E) or in the white matter around blood vessels (Figure 2F). In the control cases, these cells were found in their normal star-shaped morphology with thin appearance and numerous extensions (Figure 2D). The morphological changes shown in the figures (Case 2 in Figure 2C; Case 3 in Figure 2E,F) are representative of all three cases.

### 3.3. DENV Targets and Replicates in Brain-Resident Cells

To extend the above analyses, we sought to investigate whether the observed neurological alterations were associated with the presence of the virus in the CNS or not. 

**Figure 3:**
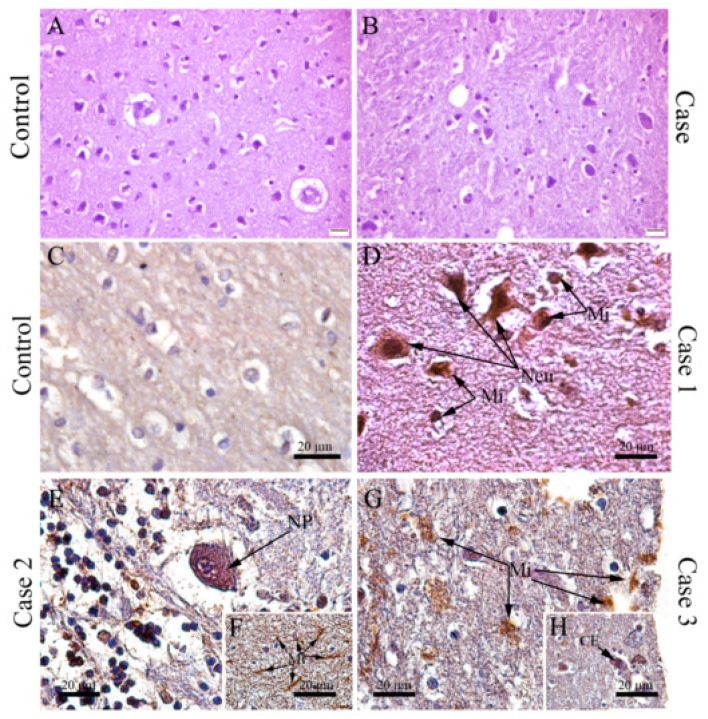
DENV antigen detection in the brain of fatal cases. (**A**) Brain tissue of a non-dengue case and (**B**) dengue case incubated only with secondary antibody; (**C**) Brain tissue of a non-dengue case incubated with primary and secondary antibodies; Detection of DENV antigen in: (**D**) neurons and microglial cells of case 1; (**E**) in Purkinje neurons (**F**) and microglia of case 2; (**G**) in microglia (**H**) and endothelial cells of case 3. Neu—Neuron, Mi—Microglia, EC—Endothelial cells, PN—Purkinje neuron.

Remarkably, DENV antigens were also detected by immunohistochemistry in the brain cuts from all three fatal dengue cases and in several distinct cell populations: in microglial cells with altered morphology (see above) (Figure 3D,F,G) and in other cell types such as Purkinje neurons (Figure 3E) and endothelial cells (Figure 3H). 

**Figure 4:**
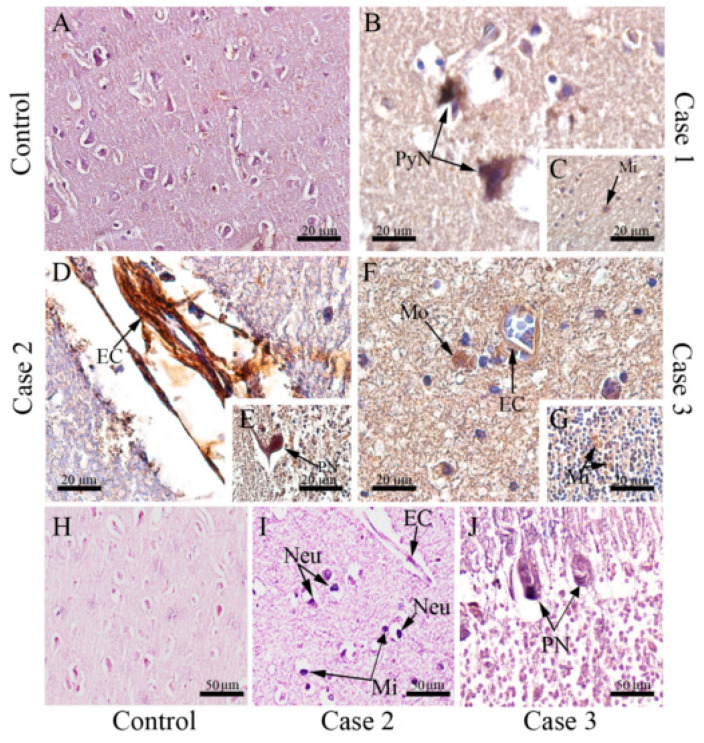
Detection of NS3 protein in the brain tissue of fatal cases. (**A**) Immunoperoxidase technique. Brain of a non-dengue case without NS3 detection; (**B**) Detection of DENV-NS3 protein in pyramidal. neurons and (**C**) microglia of the white matter in case 1; (**D**) in endothelial cells and (**E**) Purkinje neuron in case 2; (**F**) in endothelial cells and macrophages (**G**) and microglia in granular layer of case 3; (**H**) Hybridization in situ technique—brain of a non-dengue case; (**I**) Detection of DENV RNA negative strand in neurons, endothelial cells, microglial cells in case 2 (**J**) and Purkinje neurons in case 3. Neu—Neuron, Mi—Microglia, EC—Endothelial cells, Mo—Macrophages, PyN—Pyramidal neurons, PN—Purkinje neuron.

To verify viral replication in brain resident cells, we proceeded with immunostaining of the NS3 protein, a non-structural viral protein which actively participates in viral replication. NS3 antigen was detected in a broad range of cell types which included pyramidal neurons (Figure 4B), endothelial cells (Figure 4D,F), Purkinje neurons (Figure 4E), macrophages (Figure 4F), and microglia (Figure 4C,G). In a third approach, we used the in situ hybridization technique to confirm DENV replication, which was assessed using a probe which anneals in a conserved region within the NS3 gene in the negative strand of DENV RNA. Through this technique, replication of the DENV RNA was evidenced in neurons, endothelial cells, microglia cells (Figure 4I), and Purkinje neurons (Figure 4J). As expected, any detection was observed in samples of non-dengue cases (Figure 3A and Figure 4A,H). 

### 3.4. DENV Infection in the CNS Is Associated with the Local Expression of Several Pro-Inflammatory Mediators

**Figure 5:**
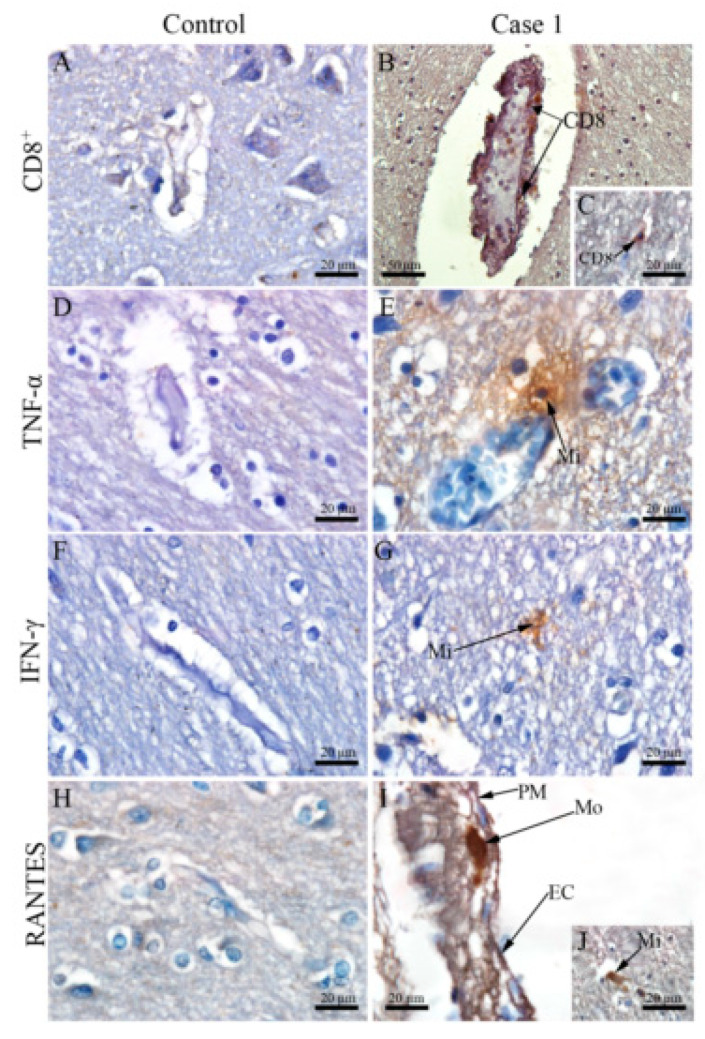
Detection of TCD8 + cells, cytokines and chemokine in brain tissue of fatal cases. (**A**,**D**,**F**,**H**) Brain tissue of a non-dengue case; (**B**) TCD8 + cells inside the capillaries and bordering them (**C**) and in the white atter; (**E**) TNF-α in microglial cells; (**G**) IFN-γ in microglial cells (**I**) RANTES in endothelial cells (**J**) and microglial cells. CD8—TCD8 + cells, Mi—Microglia, EC—Endothelial cells.

To gain insights into how the host reacts upon DENV infection in the CNS, we investigated the potential local expression of pro-inflammatory mediators. Immunohistochemistry assays revealed several TNF-α^+^- and IFN-γ^+^-microglial cells in the white matter (Figure 5E,G), which characterized a pro-inflammatory status in the CNS environment. Production of RANTES/CCL5, a chemokine associated with altered vascular permeability in dengue [41,42], was found mainly in endothelial (Figure 5I) and microglial cells (Figure 5J). Remarkably, a close-up view of the lymphocytes composing inflammatory infiltrates showed that these cells were predominantly CD8^+^ cells. CD8^+^ cells were found in nearby capillary vessels, inside and transposing these structures (Figure 5B), as well as within the white matter region (Figure 5C). The control cases did not exhibit considerable detections of CD8 cells (Figure 5A) and the cytokines cited here (Figure 5D,F,H). 

**Figure 6:**
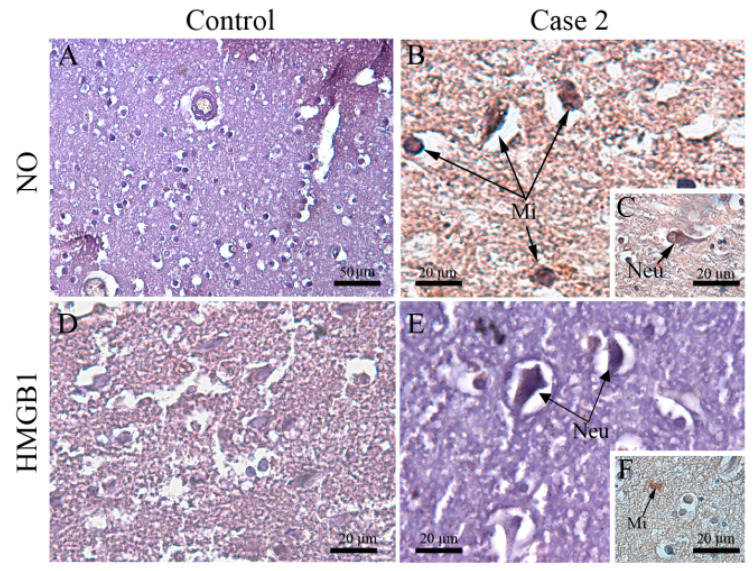
Detection of NO and HMGB1 in brain tissue of fatal cases. (**A**–**D**) Brain tissue of a non-denguecase; (**B**) Detection of NO in microglial cells (**C**) and neurons, (**E**) Detection of HMGB1 in neurons; (**F**) microglial cells. Neu—Neuron, Mi—Microglia.

Due to the previous association between nitric oxide (NO) and high mobility group box 1 (HMGB1) in severe dengue [30,43], these two pro-inflammatory markers were also considered in our investigation. Unlike control (Figure 6A), in cases 2 and 3, NO-producing microglial and neuronal cells were distributed in focal areas within the cerebral cortex (Figure 6B,C). In these cases, neurons (Figure 6E), and microglia present in the white matter were also positive for HMGB1 in their cytoplasmic region (Figure 6F), which was not seen in control (Figure 6D). 

**Figure 7:**
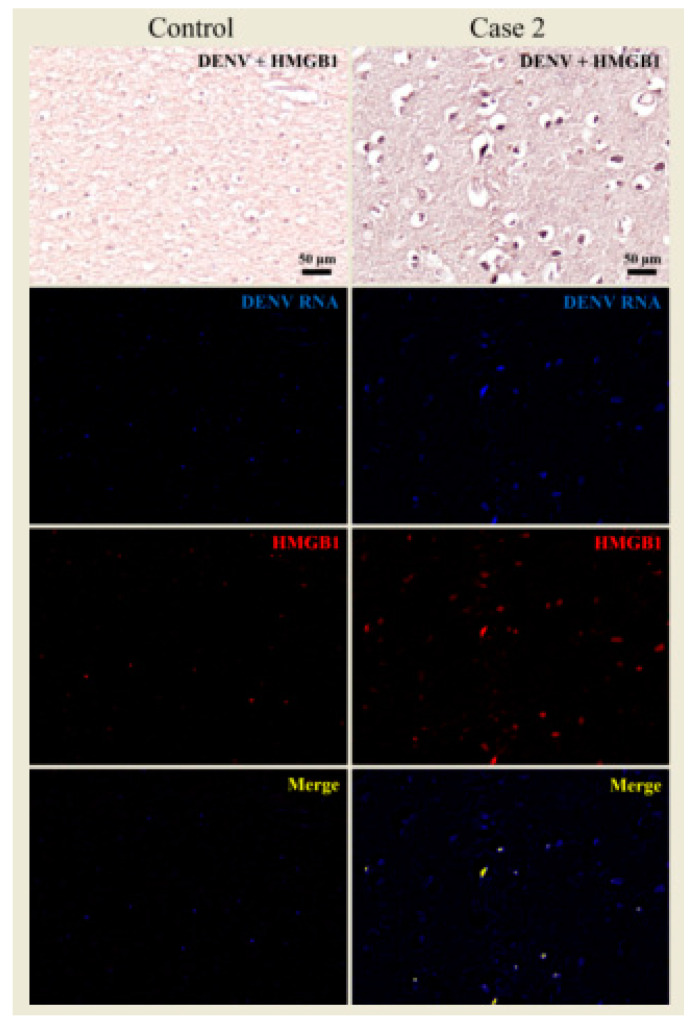
Co-staining of DENV and HMGB1 in the brain tissue of fatal case 2. In the dengue case, viral RNA was found throughout the neurons, while the expression of HMGB1 occurred within the nucleus as well as the cytoplasmic regions. DENV RNA and HMGB1 signals co-stained in the periphery and in the nucleus of the neurons. In the control case, the HMGB1 expression was marginal and predominantly in nuclear regions.

To verify whether the HMGB1 response was occurring in a DENV-specific manner, HMGB1 protein and DENV genome were co-stained combining immunohistochemistry and in situ hybridization techniques. Under this approach, the cortical region from case 2 showed degenerated neurons and microglial cells that were both positive for DENV genome or HMGB1. By merging the two signals derived from DENV and HMGB1 staining, the co-localization of these two markers was clearly seen within the cell cytoplasm of degenerated neurons and microglial cells (Figure 7). This analysis indicated a specific contribution of DENV towards a local HMGB1 pro-inflammatory response. 

## 4. Discussion

The increase in the incidence of dengue neurological cases supported the claim of neurovirulence of dengue viruses. However, the knowledge about dengue virus neuropathogenesis still has several gaps. On this sense, here we investigated the brain tissue of fatal dengue cases obtained from a dengue outbreak that occurred in Rio de Janeiro, Brazil in 2002, in which 99% of cases were caused by DENV-3 [44]. In two cases, DENV-3 was detected by RT-PCR (in brain, spleen, liver and lung samples). However, we do not have scientific information to confirm the serotype in case 1. These cases revealed brain histopathological damage, such as edema and hemorrhage, which were also reported in other rare studies with dengue post-mortem samples [45,46,47,48]. In the cortical area, we detected neurons which had lost their normal pyramidal morphology, assuming a degenerated form. These degenerated cells were also positive for NS3 protein, suggesting that DENV could directly infect neurons and actively replicate within the CNS; then, the observed CNS alterations occurred, at least partly in consequence of the direct infection of neurons by DENV. However, the neuropathological findings are not specific of dengue infection. Remarkably, we observed areas of demyelination, which are also commonly found in cases of multiple sclerosis [45]. Demyelinating neuropathy was hypothesized to be present in other arboviral diseases, such as chikungunya and zika. Infection by these viruses, in a complicated context, has been associated with the occurrence of Guillain-Barré Syndrome, which is a demyelinating disease [46,47,48].

Concerning the analyzed neuroglial cell populations (microglia and astrocytes), we found that the number of these cells were relevantly increased in the brain of fatal dengue cases, when compared to controls. This observation suggests proliferation and migration of these cells in response to the infection. In a normal situation, microglia exhibits a ramified morphology. Otherwise, upon activation their extensions tend to retract, leading to an overall amoeboid shap [49] as we observed in the fatal cases analyzed here. Astrocytes may undergo hypertrophy of their extensions [50], which is a frequent morphological change observed in cases of hypoxia [51,52]. Similar morphological alterations of glial cells were also identified in a previous study from our group under an in vivo context [53]. These divergences in morphology can suggest activation of these cell subpopulations upon DENV infection. 

The anti-DENV antibody used for immunohistochemical reactions was produced in mice inoculated with DENV-3, however, we cannot confirm, from this antibody, infection by the serotype 3, only DENV infection (by one of the four serotypes). Regarding the anti-NS3 antibody used in our assays, it was produced in mice inoculated with DENV2. However cross-reaction with other dengue serotypes [54] and other flavivirus [55,56] may occur, due to NS3 protein similarities. Therefore, here we confirm it was detected NS3 from DENV, and not NS3 from DENV-2. In addition, cross-reactivity tests were not performed for other neurotropic flaviviruses, such as West Nile virus or Zika virus; since these viruses did not circulate in Brazil in 2002.

Other reports have also shown the detection of DENV antigens in the brain in fatal cases [12,57]; however, viral replication was not assessed in this context. In the in situ hybridization technique, NS3 antigen detection and RNA negative strand were observed in distinct cell types, such as neurons, endothelial cells, Purkinje neurons, and microglia. This fact evidenced the permissiveness of different cell populations for DENV infection/expansion within the CNS, since NS3 is a non-structural protein that actively participates in the viral replication process.

Another point of discussion entails the host response to the viral presence in the CNS. A previous report from our group evidenced a link between exacerbated host immunity and tissue damages in fatal dengue cases [25,26]. Based on these previous observations, here we investigated the expression of pro-inflammatory cytokines, such as TNF-α and IFN-γ, in the CNS due to their known importance in the immunopathogenesis of the disease [55,56,57]. We detected TNF-α and IFN-γ production in microglial cells, which probably occurred in response to the viral infection in the brain. These observations corroborated with previous in-vitro experiments [58,59]. We hypothesize that a dysregulated cytokine response leads to increased vascular permeability of the blood brain-barrier (BBB) allowing viral entry into the brain parenchyma [60,61]. Such BBB dysfunction could be initially triggered by a hyper-production of pro-inflammatory cytokines in peripheral organs, which was observed in previous works [25,26]. Once viral particles enter the CNS environment, a secondary wave of production/release of pro-inflammatory cytokines within the brain would enhance BBB dysfunction, increasing viral entry into the CNS [61]. This idea is supported by the fact that several CNS endothelial cells were positive for RANTES/CCL5, which is related to permeability change. Endothelial cells were reported involved in virus entry [62,63]. BBB dysfunction was also reported in infections by several other flaviviruses, such as Japanese encephalitis virus, West Nile virus, Zika virus, yellow fever virus, and tick-borne encephalitis virus, under both in vitro and in vivo contexts [64]. Additionally, other reports have shown that changes observed in astrocytes and microglia can also impact the BBB selectivity [58,60].

Besides pro-inflammatory cytokines, we found neurons and microglia producing nitric oxide (NO) in the CNS environment of fatal dengue cases. This molecule acts in vasodilatation, neurotransmission, and host defense mechanisms in the CNS [61,62]. In low levels, NO promotes neuroinflammation, to counteract an invading pathogen or eliminate dead or damaged cells [63,64], however, in high levels NO can be directly toxic to neurons [65,66]. In viral infections, IFN-γ induces iNOS (inducible nitric oxide synthase) expression, leading to the increased production of NO [67]. There is evidence suggesting that NO competes with oxygen at cytochrome oxidase and may alter the mitochondrial energy dynamics, leading to neuronal cellular death, due to the inability to eliminate reactive oxygen species (ROS) [66,67]. The characterization of NO-producing cells in the CNS of fatal dengue cases goes in line with the histopathological findings, which exhibited neurons with degenerated aspects and signs of apoptosis [67]. In other viral diseases, as herpes encephalitis, microglial cells are the main sources of NO [68], a fact that suggests a potential role of NO-producing microglial cells in dengue neuropathogenesis.

In a previous report from our group, the same dengue cases analyzed here revealed a DENV-specific HMGB1 response in peripheral organs [30]. In this work, we observed that this response was extended to the CNS, as we detected co-localization of HMGB1 and DENV RNA in the cytoplasm of neurons and microglia. HMGB1 interacts with DNA structures in the nucleus, participating in transcription, replication, recombination, DNA repair and genomic stability [69]. In the context of cell death, such as necrosis and apoptosis, HMGB1 is released and reaches the extracellular environment, being recognized by the host’s immune system as a damage-associated molecular pattern (DAMP). These molecules comprise a class of host biomolecules that can signal to initiate and perpetuate an inflammatory response, activating the immune system [70,71]. Allonso and coworkers demonstrated that circulating levels of HMGB1 are significantly increased in DENV-infected patients, mainly during the symptomatic phase and in secondary infections [72]. Increased levels of endogenous HMGB1 have also been detected in neurodegenerative diseases, such as Alzheimer’s disease, Parkinson’s disease and multiple sclerosis, contributing to chronic neurodegeneration and progression of neuroinflammation [73]. In West Nile virus disease, HMGB1 was suggested to be detrimental thorough by mediating exacerbated inflammation [53]. In this regard, HMGB1 could contribute to the immunopathogenesis of dengue, enhancing CNS symptoms.

In conclusion, the study of the brain post-mortem samples provided valuable information about the neurotropism of DENV in the context of the severe disease. DENV was found to gain access to the brain parenchyma and to replicate into endothelial cells, neurons, and microglia. It is known that not all dengue infections lead to neurological manifestations; however, in the small number of cases that DENV correlates with CNS pathology; it is possible that the virus activates glial cells triggering a harmful local immune response. This response could be guided by an array of pro-inflammatory mediators such as TNF-α, IFN-γ, NO and HMGB1. This work shed light on the neuropathogenesis of dengue, highlighting possible inflammatory markers that could play a role in the CNS-related symptomatology.
